# {4-Dimethyl­amino-*N*′-[1-(2-oxidophen­yl)ethyl­idene]benzohydrazidato}(methano­lato)oxidovanadium(V)

**DOI:** 10.1107/S1600536811050173

**Published:** 2011-11-25

**Authors:** Chen-Yi Wang, Juan-Juan Hu, Hai-Yu Tu, Pei-Fei Zhu, Su-Jun Sheng

**Affiliations:** a Department of Chemistry, Huzhou University, Huzhou 313000, People’s Republic of China; b Huzhou No. 11 Middle School, Huzhou 313000, People’s Republic of China

## Abstract

The title oxidovanadium(V) complex, [V(C_17_H_17_N_3_O_2_)(CH_3_O)O], was obtained by the reaction of 2-acetyl­phenol, 4-dimethyl­amino­benzohydrazide and vanadyl sulfate in methanol. The V^V^ atom is five-coordinated by *N*,*O*,*O*′-donor atoms of the Schiff base ligand, one meth­oxy O atom and one oxide O atom, forming a square-pyramidal geometry.

## Related literature

For Schiff base complexes, see: Wang (2009 ▶); Wang & Ye (2011 ▶). For similar oxidovanadium complexes, see: Deng *et al.* (2005 ▶); Gao *et al.* (2005 ▶); Huo *et al.* (2004 ▶).
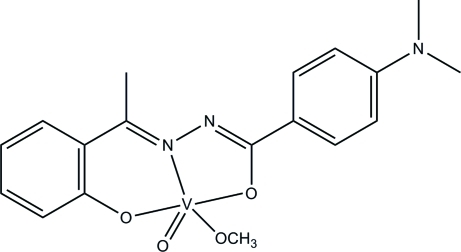


## Experimental

### 

#### Crystal data

[V(C_17_H_17_N_3_O_2_)(CH_3_O)O]
*M* *_r_* = 393.31
Monoclinic, 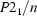

*a* = 7.4670 (15) Å
*b* = 16.769 (3) Å
*c* = 14.301 (3) Å
β = 97.317 (3)°
*V* = 1776.1 (6) Å^3^
*Z* = 4
Mo *K*α radiation
μ = 0.59 mm^−1^
*T* = 298 K
0.37 × 0.35 × 0.32 mm

#### Data collection

Bruker SMART CCD area-detector diffractometer
Absorption correction: multi-scan (*SADABS*; Sheldrick, 1996 ▶) *T*_min_ = 0.812, *T*_max_ = 0.834
14014 measured reflections
3784 independent reflections
2320 reflections with *I* > 2σ(*I*)
*R*_int_ = 0.086

#### Refinement

*R*[*F* ^2^ > 2σ(*F* ^2^)] = 0.083
*wR*(*F* ^2^) = 0.185
*S* = 1.04
3784 reflections
239 parameters
H-atom parameters constrained
Δρ_max_ = 0.41 e Å^−3^
Δρ_min_ = −0.35 e Å^−3^

### 

Data collection: *SMART* (Bruker, 1998 ▶); cell refinement: *SAINT* (Bruker, 1998 ▶); data reduction: *SAINT*; program(s) used to solve structure: *SHELXS97* (Sheldrick, 2008 ▶); program(s) used to refine structure: *SHELXL97* (Sheldrick, 2008 ▶); molecular graphics: *SHELXTL* (Sheldrick, 2008 ▶); software used to prepare material for publication: *SHELXTL*.

## Supplementary Material

Crystal structure: contains datablock(s) global, I. DOI: 10.1107/S1600536811050173/qm2035sup1.cif

Structure factors: contains datablock(s) I. DOI: 10.1107/S1600536811050173/qm2035Isup2.hkl

Additional supplementary materials:  crystallographic information; 3D view; checkCIF report

## Figures and Tables

**Table 1:** Selected bond lengths (Å)

V1—O2	1.584 (4)
V1—O3	1.781 (3)
V1—O1	1.830 (3)
V1—O4	1.891 (3)
V1—N1	2.087 (4)

**Fig. 1.**
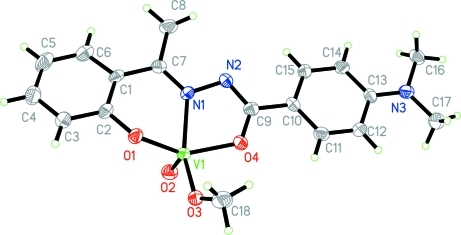
The molecular structure of the title compound, showing the atom-numbering scheme. Displacement ellipsoids are drawn at the 30% probability level.

## supplementary crystallographic information

### Comment

As part of our investigations into new Schiff base complexes (Wang & Ye, 2011; Wang, 2009), we have synthesized the title compound, a new mononuclear oxovanadium(V) complex, Fig. 1. The V atom in the complex is five-coordinated by the NOO donor atoms of the Schiff base ligand, one methoxy O atom, and one oxo O atom, forming a square pyramidal geometry. The V–O and V–N bond lengths (Table 1) are typical and are comparable with those observed in other similar vanadium complexes (Deng *et al.*, 2005; Gao *et al.*, 2005; Huo *et al.*, 2004). The dihedral angle between the two benzene rings is 9.6 (3)°.

### Experimental

2-Acetylphenol (1.0 mmol, 0.14 g), 4-dimethylaminobenzohydrazide (1.0 mmol, 0.18 g), and vanadyl sulfate (1.0 mmol, 0.16 g) were dissolved in methanol (30 ml). The mixture was stirred at room temperature for 10 min to give a clear brown solution. After keeping the solution in air for a week, brown block-shaped crystals were formed at the bottom of the vessel.

### Refinement

All H atoms were placed in geometrically idealized positions and constrained to ride on their parent atoms, with C—H distances in the range 0.93–0.96 Å, and with *U*_iso_(H) set at 1.2 or 1.5*U*_eq_(C).

### Crystal data

**Table:**

[V(C_17_H_17_N_3_O_2_)(CH_3_O)O]	*F*(000) = 816
*M**_r_* = 393.31	*D*_x_ = 1.471 Mg m^−^^3^
Monoclinic, *P*2_1_/*n*	Mo *K*α radiation, λ = 0.71073 Å
*a* = 7.4670 (15) Å	Cell parameters from 1094 reflections
*b* = 16.769 (3) Å	θ = 2.5–24.5°
*c* = 14.301 (3) Å	µ = 0.59 mm^−^^1^
β = 97.317 (3)°	*T* = 298 K
*V* = 1776.1 (6) Å^3^	Block, brown
*Z* = 4	0.37 × 0.35 × 0.32 mm

### Data collection

**Table:**

Bruker SMART CCD area-detector diffractometer	3784 independent reflections
Radiation source: fine-focus sealed tube	2320 reflections with *I* > 2σ(*I*)
graphite	*R*_int_ = 0.086
ω scan	θ_max_ = 27.0°, θ_min_ = 1.9°
Absorption correction: multi-scan (*SADABS*; Sheldrick, 1996)	*h* = −9→9
*T*_min_ = 0.812, *T*_max_ = 0.834	*k* = −21→21
14014 measured reflections	*l* = −17→17

### Refinement

**Table:**

Refinement on *F*^2^	Primary atom site location: structure-invariant direct methods
Least-squares matrix: full	Secondary atom site location: difference Fourier map
*R*[*F*^2^ > 2σ(*F*^2^)] = 0.083	Hydrogen site location: inferred from neighbouring sites
*wR*(*F*^2^) = 0.185	H-atom parameters constrained
*S* = 1.04	*w* = 1/[σ^2^(*F*_o_^2^) + (0.0631*P*)^2^ + 2.2577*P*] where *P* = (*F*_o_^2^ + 2*F*_c_^2^)/3
3784 reflections	(Δ/σ)_max_ = 0.001
239 parameters	Δρ_max_ = 0.41 e Å^−^^3^
0 restraints	Δρ_min_ = −0.35 e Å^−^^3^

### Special details

**Table:**

Geometry. All e.s.d.'s (except the e.s.d. in the dihedral angle between two l.s. planes) are estimated using the full covariance matrix. The cell e.s.d.'s are taken into account individually in the estimation of e.s.d.'s in distances, angles and torsion angles; correlations between e.s.d.'s in cell parameters are only used when they are defined by crystal symmetry. An approximate (isotropic) treatment of cell e.s.d.'s is used for estimating e.s.d.'s involving l.s. planes.
Refinement. Refinement of *F*^2^ against ALL reflections. The weighted *R*-factor *wR* and goodness of fit *S* are based on *F*^2^, conventional *R*-factors *R* are based on *F*, with *F* set to zero for negative *F*^2^. The threshold expression of *F*^2^ > σ(*F*^2^) is used only for calculating *R*-factors(gt) *etc*. and is not relevant to the choice of reflections for refinement. *R*-factors based on *F*^2^ are statistically about twice as large as those based on *F*, and *R*- factors based on ALL data will be even larger.

### Fractional atomic coordinates and isotropic or equivalent isotropic displacement parameters (Å^2^)

**Table:**

	*x*	*y*	*z*	*U*_iso_*/*U*_eq_	
V1	0.10795 (12)	0.13127 (5)	0.77749 (6)	0.0388 (3)	
N1	0.1980 (5)	0.1517 (2)	0.9197 (3)	0.0358 (9)	
N2	0.2327 (6)	0.0829 (2)	0.9744 (3)	0.0408 (10)	
N3	0.3041 (7)	−0.2889 (3)	1.0718 (3)	0.0524 (12)	
O1	0.0163 (5)	0.23203 (18)	0.7837 (2)	0.0412 (9)	
O2	0.2945 (5)	0.1411 (2)	0.7370 (2)	0.0504 (9)	
O3	−0.0496 (5)	0.0947 (2)	0.6833 (2)	0.0492 (10)	
O4	0.1059 (5)	0.02996 (19)	0.8355 (2)	0.0461 (9)	
C1	0.1993 (7)	0.2944 (3)	0.9142 (3)	0.0384 (12)	
C2	0.0922 (7)	0.2980 (3)	0.8249 (4)	0.0403 (12)	
C3	0.0610 (7)	0.3703 (3)	0.7791 (4)	0.0492 (13)	
H3	−0.0122	0.3726	0.7215	0.059*	
C4	0.1378 (8)	0.4385 (3)	0.8187 (5)	0.0592 (16)	
H4	0.1179	0.4868	0.7871	0.071*	
C5	0.2445 (9)	0.4365 (4)	0.9049 (5)	0.0658 (18)	
H5	0.2977	0.4830	0.9309	0.079*	
C6	0.2712 (8)	0.3661 (3)	0.9516 (4)	0.0555 (15)	
H6	0.3400	0.3657	1.0106	0.067*	
C7	0.2311 (7)	0.2191 (3)	0.9642 (3)	0.0402 (12)	
C8	0.3029 (8)	0.2198 (3)	1.0662 (4)	0.0557 (15)	
H8A	0.2411	0.1803	1.0987	0.084*	
H8B	0.2842	0.2715	1.0921	0.084*	
H8C	0.4297	0.2080	1.0736	0.084*	
C9	0.1847 (7)	0.0219 (3)	0.9224 (3)	0.0397 (12)	
C10	0.2147 (7)	−0.0592 (3)	0.9614 (3)	0.0393 (12)	
C11	0.1577 (7)	−0.1257 (3)	0.9080 (3)	0.0446 (13)	
H11	0.0989	−0.1189	0.8472	0.053*	
C12	0.1870 (7)	−0.2012 (3)	0.9435 (4)	0.0444 (13)	
H12	0.1489	−0.2448	0.9059	0.053*	
C13	0.2734 (7)	−0.2142 (3)	1.0356 (3)	0.0396 (12)	
C14	0.3301 (7)	−0.1464 (3)	1.0886 (3)	0.0434 (13)	
H14	0.3884	−0.1525	1.1496	0.052*	
C15	0.3009 (7)	−0.0714 (3)	1.0520 (3)	0.0443 (13)	
H15	0.3400	−0.0275	1.0888	0.053*	
C16	0.3821 (8)	−0.3013 (3)	1.1697 (4)	0.0532 (15)	
H16A	0.4991	−0.2768	1.1802	0.080*	
H16B	0.3934	−0.3574	1.1822	0.080*	
H16C	0.3050	−0.2778	1.2109	0.080*	
C17	0.2427 (9)	−0.3588 (3)	1.0172 (4)	0.0625 (17)	
H17A	0.1134	−0.3619	1.0116	0.094*	
H17B	0.2941	−0.4058	1.0483	0.094*	
H17C	0.2800	−0.3551	0.9555	0.094*	
C18	−0.2125 (9)	0.0551 (4)	0.6885 (4)	0.0698 (19)	
H18A	−0.1942	0.0135	0.7349	0.105*	
H18B	−0.2551	0.0322	0.6282	0.105*	
H18C	−0.3003	0.0922	0.7059	0.105*	

### Atomic displacement parameters (Å^2^)

**Table:**

	*U*^11^	*U*^22^	*U*^33^	*U*^12^	*U*^13^	*U*^23^
V1	0.0510 (6)	0.0343 (5)	0.0295 (4)	0.0004 (4)	−0.0007 (4)	−0.0003 (4)
N1	0.037 (2)	0.033 (2)	0.036 (2)	0.0009 (18)	0.0023 (18)	−0.0049 (17)
N2	0.050 (3)	0.038 (2)	0.034 (2)	0.002 (2)	0.0012 (19)	0.0052 (19)
N3	0.076 (3)	0.044 (3)	0.037 (2)	0.006 (2)	0.007 (2)	0.000 (2)
O1	0.047 (2)	0.0345 (19)	0.0398 (19)	0.0037 (16)	−0.0043 (16)	−0.0023 (15)
O2	0.056 (2)	0.051 (2)	0.045 (2)	0.0061 (18)	0.0107 (18)	−0.0004 (17)
O3	0.067 (3)	0.040 (2)	0.037 (2)	−0.0089 (19)	−0.0069 (18)	0.0006 (16)
O4	0.063 (2)	0.039 (2)	0.0331 (19)	0.0004 (18)	−0.0069 (17)	0.0053 (15)
C1	0.039 (3)	0.039 (3)	0.040 (3)	−0.003 (2)	0.014 (2)	−0.015 (2)
C2	0.039 (3)	0.035 (3)	0.049 (3)	0.002 (2)	0.010 (2)	−0.009 (2)
C3	0.057 (4)	0.042 (3)	0.050 (3)	−0.001 (3)	0.009 (3)	−0.003 (3)
C4	0.068 (4)	0.043 (3)	0.070 (4)	0.002 (3)	0.021 (3)	0.000 (3)
C5	0.069 (4)	0.047 (4)	0.084 (5)	−0.020 (3)	0.018 (4)	−0.023 (3)
C6	0.056 (4)	0.053 (4)	0.059 (4)	−0.007 (3)	0.011 (3)	−0.017 (3)
C7	0.042 (3)	0.044 (3)	0.036 (3)	0.000 (2)	0.008 (2)	−0.003 (2)
C8	0.062 (4)	0.062 (4)	0.040 (3)	−0.002 (3)	−0.005 (3)	−0.012 (3)
C9	0.034 (3)	0.047 (3)	0.040 (3)	0.000 (2)	0.012 (2)	−0.004 (2)
C10	0.041 (3)	0.042 (3)	0.035 (3)	0.002 (2)	0.007 (2)	0.009 (2)
C11	0.046 (3)	0.052 (3)	0.034 (3)	−0.003 (3)	0.001 (2)	0.000 (3)
C12	0.051 (3)	0.041 (3)	0.041 (3)	−0.007 (3)	0.004 (3)	−0.005 (2)
C13	0.043 (3)	0.038 (3)	0.039 (3)	0.002 (2)	0.011 (2)	0.007 (2)
C14	0.048 (3)	0.047 (3)	0.032 (3)	0.006 (2)	−0.006 (2)	0.002 (2)
C15	0.053 (3)	0.041 (3)	0.038 (3)	−0.004 (3)	0.001 (2)	0.000 (2)
C16	0.064 (4)	0.052 (3)	0.044 (3)	0.013 (3)	0.008 (3)	0.013 (3)
C17	0.083 (5)	0.050 (4)	0.056 (4)	0.006 (3)	0.010 (3)	0.002 (3)
C18	0.082 (5)	0.075 (4)	0.050 (4)	−0.021 (4)	−0.005 (3)	−0.009 (3)

### Geometric parameters (Å, °)

**Table:**

V1—O2	1.584 (4)	C7—C8	1.489 (7)
V1—O3	1.781 (3)	C8—H8A	0.9600
V1—O1	1.830 (3)	C8—H8B	0.9600
V1—O4	1.891 (3)	C8—H8C	0.9600
V1—N1	2.087 (4)	C9—C10	1.477 (7)
N1—C7	1.305 (6)	C10—C15	1.386 (7)
N1—N2	1.399 (5)	C10—C11	1.387 (7)
N2—C9	1.289 (6)	C11—C12	1.372 (7)
N3—C13	1.363 (6)	C11—H11	0.9300
N3—C17	1.451 (7)	C12—C13	1.408 (7)
N3—C16	1.461 (6)	C12—H12	0.9300
O1—C2	1.343 (5)	C13—C14	1.401 (7)
O3—C18	1.396 (7)	C14—C15	1.369 (7)
O4—C9	1.312 (6)	C14—H14	0.9300
C1—C6	1.395 (7)	C15—H15	0.9300
C1—C2	1.420 (7)	C16—H16A	0.9600
C1—C7	1.456 (7)	C16—H16B	0.9600
C2—C3	1.384 (7)	C16—H16C	0.9600
C3—C4	1.369 (7)	C17—H17A	0.9600
C3—H3	0.9300	C17—H17B	0.9600
C4—C5	1.381 (8)	C17—H17C	0.9600
C4—H4	0.9300	C18—H18A	0.9600
C5—C6	1.358 (8)	C18—H18B	0.9600
C5—H5	0.9300	C18—H18C	0.9600
C6—H6	0.9300		
			
O2—V1—O3	106.64 (18)	H8A—C8—H8B	109.5
O2—V1—O1	105.91 (17)	C7—C8—H8C	109.5
O3—V1—O1	98.04 (16)	H8A—C8—H8C	109.5
O2—V1—O4	108.07 (17)	H8B—C8—H8C	109.5
O3—V1—O4	88.89 (15)	N2—C9—O4	121.5 (5)
O1—V1—O4	141.65 (16)	N2—C9—C10	119.7 (5)
O2—V1—N1	98.64 (17)	O4—C9—C10	118.8 (4)
O3—V1—N1	153.28 (17)	C15—C10—C11	118.0 (5)
O1—V1—N1	82.92 (15)	C15—C10—C9	121.3 (5)
O4—V1—N1	74.93 (14)	C11—C10—C9	120.7 (5)
C7—N1—N2	115.5 (4)	C12—C11—C10	121.0 (5)
C7—N1—V1	129.4 (3)	C12—C11—H11	119.5
N2—N1—V1	115.0 (3)	C10—C11—H11	119.5
C9—N2—N1	108.3 (4)	C11—C12—C13	121.4 (5)
C13—N3—C17	120.8 (4)	C11—C12—H12	119.3
C13—N3—C16	121.5 (4)	C13—C12—H12	119.3
C17—N3—C16	117.4 (4)	N3—C13—C14	121.0 (5)
C2—O1—V1	129.9 (3)	N3—C13—C12	122.1 (5)
C18—O3—V1	128.3 (3)	C14—C13—C12	116.9 (4)
C9—O4—V1	118.7 (3)	C15—C14—C13	121.1 (5)
C6—C1—C2	117.0 (5)	C15—C14—H14	119.5
C6—C1—C7	121.9 (5)	C13—C14—H14	119.5
C2—C1—C7	121.1 (4)	C14—C15—C10	121.6 (5)
O1—C2—C3	118.6 (5)	C14—C15—H15	119.2
O1—C2—C1	121.2 (4)	C10—C15—H15	119.2
C3—C2—C1	120.2 (5)	N3—C16—H16A	109.5
C4—C3—C2	120.0 (5)	N3—C16—H16B	109.5
C4—C3—H3	120.0	H16A—C16—H16B	109.5
C2—C3—H3	120.0	N3—C16—H16C	109.5
C3—C4—C5	120.9 (6)	H16A—C16—H16C	109.5
C3—C4—H4	119.6	H16B—C16—H16C	109.5
C5—C4—H4	119.6	N3—C17—H17A	109.5
C6—C5—C4	119.5 (5)	N3—C17—H17B	109.5
C6—C5—H5	120.3	H17A—C17—H17B	109.5
C4—C5—H5	120.3	N3—C17—H17C	109.5
C5—C6—C1	122.3 (6)	H17A—C17—H17C	109.5
C5—C6—H6	118.8	H17B—C17—H17C	109.5
C1—C6—H6	118.8	O3—C18—H18A	109.5
N1—C7—C1	120.2 (4)	O3—C18—H18B	109.5
N1—C7—C8	120.5 (5)	H18A—C18—H18B	109.5
C1—C7—C8	119.3 (5)	O3—C18—H18C	109.5
C7—C8—H8A	109.5	H18A—C18—H18C	109.5
C7—C8—H8B	109.5	H18B—C18—H18C	109.5
